# Pilot implementation of a telehealth safety planning group intervention for suicidal rural Veterans enhanced by lived experience veteran peer participation

**DOI:** 10.3389/fpsyt.2025.1512523

**Published:** 2025-03-18

**Authors:** Marianne Goodman, Madison Strouse, Caroline Boucher, Sofie Glatt, James Jacobs, Angie Waliski, Emilia Fonseca, Terra Osterberg, Sapana Patel

**Affiliations:** ^1^ VISN 2 Mental Illness Research, Education and Clinical Center, James J. Peters Veterans Affairs Medical Center, Bronx, NY, United States; ^2^ Department of Psychiatry, Icahn School of Medicine at Mount Sinai, New York, NY, United States; ^3^ We are the 22, Little Rock, AR, United States; ^4^ Center for Mental Healthcare and Outcomes Research, Center for Advancing Community-engaged Research and Evaluation in Suicide Prevention, Central Arkansas Veterans Healthcare System, North Little Rock, AR, United States; ^5^ Department of Psychiatry, University of Arkansas for Medical Science, Little Rock, AR, United States; ^6^ Department of Psychology, University of Montana College of Humanities and Sciences, Missoula, MT, United States; ^7^ Center for Excellence in Cultural Competence, The New York State Psychiatric Institute, New York, NY, United States; ^8^ Department of Psychiatry, Columbia University Vagelos College of Physicians and Surgeons, New York, NY, United States

**Keywords:** telehealth, group therapy, peer, safety planning, lethal means safety

## Abstract

Despite the Veterans Administration (VA) designating suicide prevention as the number one clinical priority, Veteran suicide rates continue to rise. One sub-population at elevated risk are Veterans living in rural communities given their heightened availability of firearms coupled with more limited access to mental health services. Telehealth delivery of treatment is a potential solution for the provision of critical services to rural areas. Despite the expansive growth of virtual treatment after the pandemic, there exist few suicide-specific telehealth health services. Our community case study aims to address this gap by piloting a manualized suicide safety planning and firearm safety group, titled Project Life Force (PLF), delivered virtually to rural Arkansas. The project’s goal was to specifically enhance rural Veteran engagement with telehealth delivery through the use of community-based lived-experience rural peers. We present the rationale and details of the PLF intervention with a focus on the community Veteran peer enhancement component. This case study presents an innovative treatment design of a group led by a clinician augmented by a peer recovery leader that facilitated detailed conversations of how to limit suicide risk, encouraged disclosure about suicide symptoms, and promoted suicide related coping including encouragement of help-seeking behavior and safer storage of firearms. While the inclusion of a peer recovery leader was felt to be instrumental to the PLF-PE group’s success, special attention to the peer recovery leader is essential and includes specific training, regular supervision as well as attention and support regarding the psychological impacts of self-disclosure and assuming a leadership role. This case study highlights the invaluable role that lived experience peers can play in suicide prevention treatment efforts and lethal means safety and paves the way for continued development of this effort.

## 1 Introduction

### 1.1 Elevated Veteran suicide and firearm suicide rates

The prevention of suicide and treatment of those at risk remains a national priority for both civilians and Veterans. The 2023 VA Suicide Report (1) highlighted that despite the Veteran’s Health Administration’s provision of enhanced suicide prevention services and support of various clinical suicide risk treatments, Veteran suicide rates continue to be 1.5 the rate in the civilian population, with approximately 17 Veterans dying by suicide every day. From 2001 through 2020, age- and sex-adjusted suicide rates of Veterans exceeded those of non-Veteran adults and in 2020, the Veteran suicide rate was 57.3% higher than that of civilians. Incidence of suicide by firearm has substantially increased in the Veteran population in the last two decades and has become the most prevalent method, exceeding the proportion of firearm suicide in the non-Veteran population (71% vs. 50.3% in 2020) (1). These statistics underscore the need for targeted suicide prevention services that support and engage geographically isolated Veterans at increased for suicide by firearm.

### 1.2 Rural Veterans, community partnerships, and veteran suicide risk

Relative to urban Veterans, rural Veterans have compounded suicide risk, stemming from constraints in access to mental and physical health care, lower quality of life, socioeconomic inequalities, education, community resources, and increased firearm ownership (2–4). Recent advances in addressing the rural Veteran suicide risk problem include programming from the 2018 launch of VA’s National Strategy for Preventing Veteran Suicide (5), which promotes a public health approach to Veteran suicide prevention and specifically aims to combine community-based suicide prevention strategies and clinically based interventions. However, there are few “suicide-specific” clinical interventions integrated into community partnerships. Moreover, there is increasing appreciation of the potential of telemental health to address the issues confronting rural suicidal populations. However, a recent review of telemental health suicide-specific treatments (6) highlights a paucity of telehealth effectiveness studies and few implementation projects.

### 1.3 Benefits of lived experience Veteran peers

There is growing recognition of the benefits of including peers (i.e., individuals with histories of success living with serious mental illness (SMI) who support others with SMI) in the provision of mental health and suicide prevention services. For Veterans with suicidal thoughts and behaviors, the benefits of peer support may be particularly potent given the importance of hopelessness, lack of connectedness, loneliness, and stigma to suicide theory (e.g., interpersonal theory of suicide; 7) and risk (7–10). Peer support can also improve suicidal Veterans‘ potentially negative experiences of treatment, through reduction of stigma around suicide disclosure and providing a unique capacity for therapeutic empathy in treatment which may otherwise be overshadowed by traditional services’ emphasis on risk (11).

The potential benefits of peer support services on suicide risk have spurred several national suicide prevention agendas to emphasize integrating peer support services into existing suicide prevention infrastructures (12, 13). The VHA has been a national leader in the development of peer support groups for Veterans with SMI leading to reduced substance use, increased treatment adherence, improved social support, and a reduction of negative symptoms (14, 15). Despite these benefits, peer support has not been fully leveraged as a suicide prevention strategy (16).

A recent scoping review by VA researchers of peer support activities, including 84 studies categorized by primary function and type of peer relationship (17), highlights the feasibility, acceptability, low cost, and flexibility of these services in mental health services broadly. The review indicated that most peer support services were conducted outside of mental health systems, in settings such as crisis lines and correctional facilities (17). While peer support was utilized to support crisis services and promote help-seeking, peer support remained critically underutilized to bolster evidence-based treatment, life skills, and lethal means safety. This review underscores the valuable role that peers may play in existing suicide prevention efforts while highlight several unexplored avenues where peers could enhance suicide prevention efforts, particularly in rural community settings.

### 1.4 Lived experience peers can play a critical role in lethal means safety counseling

Lethal means safety counseling (LMSC) promotes safe storage of firearms, especially during times of increased risk, with a recent emphasis on suicide prevention practice for Veterans and active-duty service personnel (18). Khazanov and colleagues (19) conducted a qualitative systematic review of studies examining stakeholder perceptions of LMSC and identified seven themes fostering its implementation. Some of the themes, including 1) stressing the importance of firearms as an identity and right, 2) fostering understanding of the rationale for LMSC, 3) helping providers demonstrate cultural competency around firearm use and, 4) involving trusted family and friends in LMSC, could be met by including firearm-owning, lived experience peers in LMSC efforts.

### 1.5 Safety planning group telehealth intervention- Project Life Force

Over the past 8 years, our team has developed and tested Project Life Force (PLF), a 10-session manualized telehealth group intervention, that utilizes dialectical behavior therapy skills to enhance both suicide safety planning and lethal means safety for high-risk suicidal Veterans (20–22). The group format is consistent with extant literature suggesting belongingness and connectedness as key facilitators of suicide risk reduction (23, 24) in addition to military-specific protective factors such as “unit cohesion” (25). PLF aims to enhance safety planning for suicidal Veterans by promoting suicide-related coping skills and social support. The telehealth modality is a promising avenue for expansion to rural communities.

### 1.6 Expansion of PLF to rural communities in Arkansas

Our team met with Arkansas stakeholders including rural Veterans, VHA peer specialists, and community-based Veteran first responders (n=10) from local Veteran organizations involved with suicidal Veterans for input on how to deliver the PLF intervention virtually for their populations. Feedback stressed the importance of including individuals with lived experience in the group as a local ally to facilitate retention in the program and provide participants with in-person support and access to local resources.

In this community case study, we describe the development and initial piloting of a PLF program for rural Veterans enhanced by lived experience rural community Veteran peers called PLF-Peer Enhancement (PLF-PE). Using qualitative interviews, we report on the experiences of participants in PLF-PE and their views on the acceptability and feasibility of this innovative VA-community partnered effort.

## 2 Context

Our team’s expansion to Arkansas was made with consideration of population factors. Arkansas has a large Veteran population, high gun ownership rates, and is largely rural with 44% of residents living in rural areas (26). The state has just over 3 million people, of whom are about 70% white with median age 38.9, and 7% are Veterans. With 57.2 percent of Arkansas residents owning guns, the state has the 6th highest firearm ownership rates in the country (27).

### 2.1 Our community partner (We are the 22)

Our community partner, WAT22, founded as a 501 (3)(c) in 2017, provides crisis services (termed “responses”) to suicidal Veterans across the state of Arkansas. The WAT22 organization began in 2018 when the reports of Veterans dying by suicide was 22 a day, hence the organizations moniker. Furthermore, the founder of WAT22, a Veteran who survived a suicide attempt, reasoned that a peer-to-peer supportive approach using Veterans as volunteers would be more effective in reaching other Veterans who might be in a suicidal crisis. The vision was to create a Veteran peer volunteer network, with a mobile outreach program designed to provide “boots on the ground” support and resources for Veterans struggling with mental health issues and suicidal ideation. There are currently 95 trained Veteran WAT22 members active and available for dispatch who have performed over 580 responses statewide with only one death by suicide post-response. Most of the responders are combat Veterans, and many have lived experience with suicide ideation or behavior. Their mission, “empowering veterans to combat veteran suicide on the front lines through peer-to-peer intervention,” aligns with the goals of PLF-PE.

## 3 Key programmatic elements of PLF- peer enhancement

### 3.1 PLF group content

The original PLF intervention (21) was designed to provide a mechanism to develop and enhance suicide safety planning over time. In PLF, Veterans revise their safety plans while learning distress tolerance, emotion regulation, and interpersonal skills to incorporate into their plans (20, 21). Additional topics include firearm and lethal means safety, augmenting physical well-being, strategies on how to share their plan with family/significant others, and how to access crisis line services.

**Figure 1:**
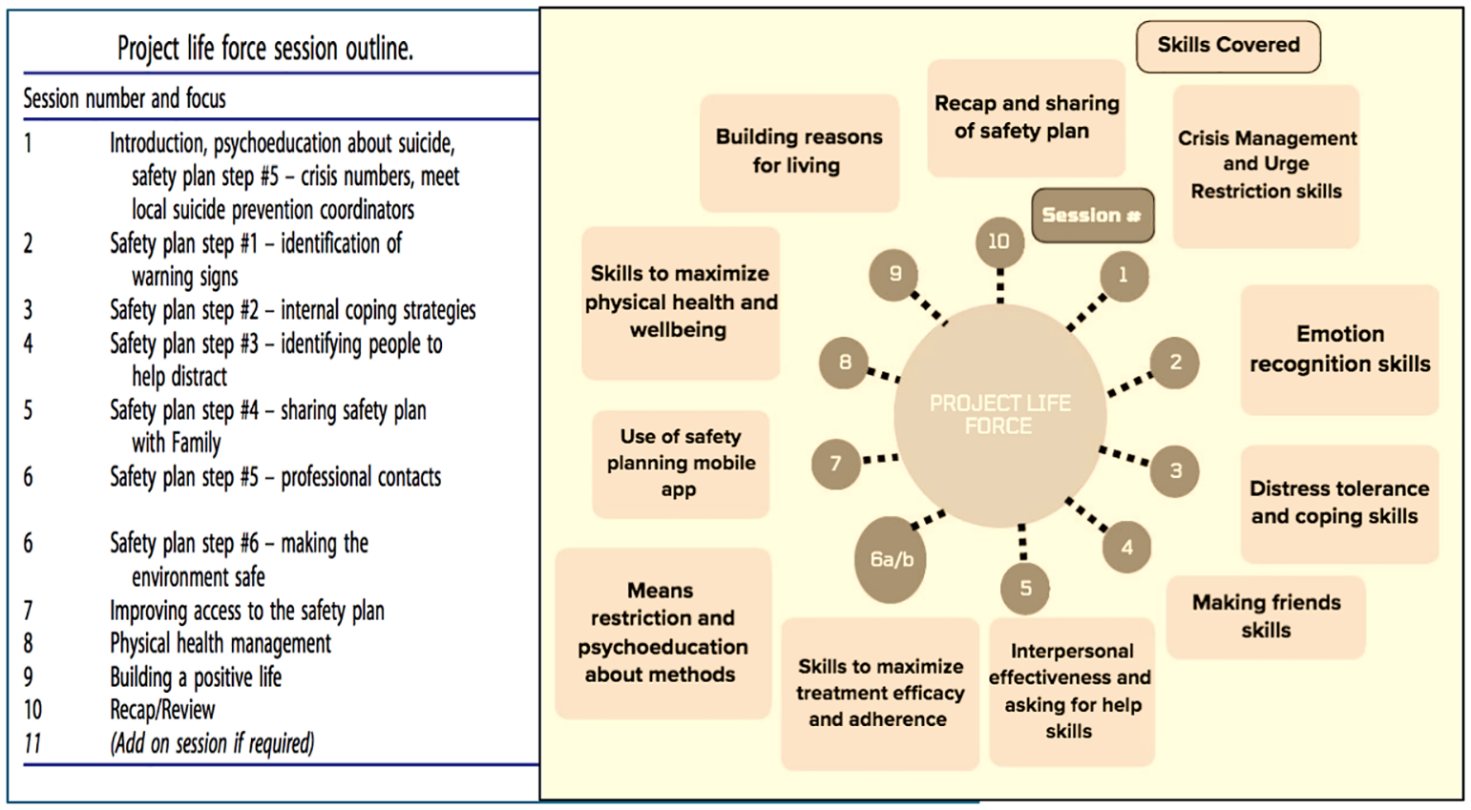
Project life force session outline.

See **Figure 1** for specific session content and PLF Skills.

### 3.2 Addition of the peer recovery leader role

**Figure 2:**
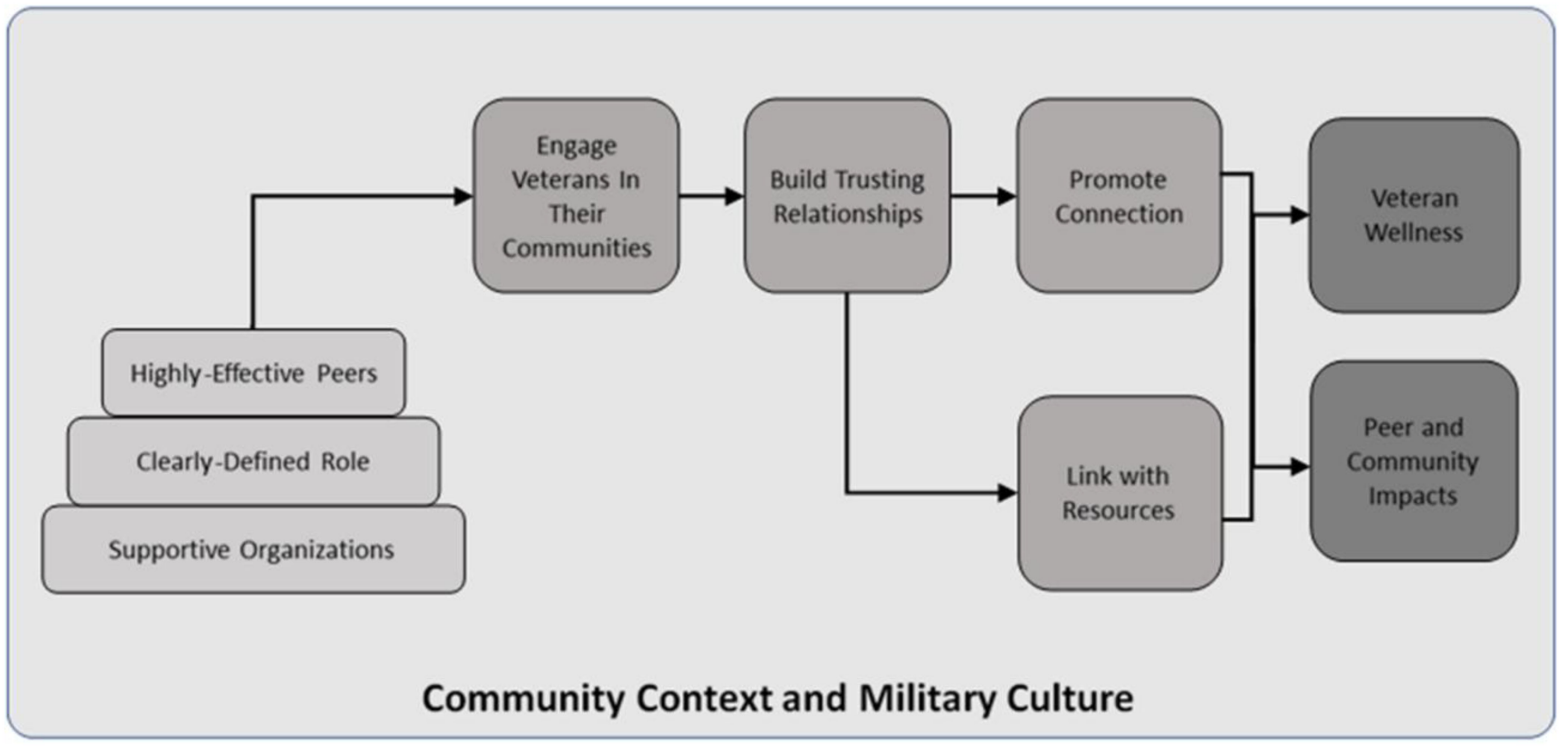
Model of community-based veteran peer suicide prevention from Beehler et al., 2021 Project Life Force-Peer Enhancement.

Our intervention adaptation, PLF-PE integrates stakeholder feedback by incorporating community-based Veteran first responders with lived experience of past suicidal crises and firearm risk as peer recovery leaders (PRL). The PRL role was defined as a Veteran with lived experience who has not had a suicide attempt in the last year, has completed one cycle of PLF as a participant, and has completed the basic training in suicide prevention (28) and PLF-specific training. Our goal was for community-based peers in recovery with lived experience of suicide behavior and firearm ownership to function as “trusted messengers,” (29) sharing their experience with improved suicide-related coping and safer storage of firearms. Additionally, we anticipated that the PRL would inspire hope and improve engagement in the virtual group intervention. The PRL’s main role is to help facilitate discussion by providing input, participating in all the group exercises, modeling homework practice and demonstrating how the PLF material can be used in their everyday life. **Figure 2** demonstrates our model of community-based Veteran lived experience peer involvement. **Table 1** outlines the specific tasks of the lived experience peer in the PLF-PE groups.

**Table 1:** Lived experience peer roles in the PLF-PE group.

Lived Experience Peer Roles:
1. model disclosure
2. share their lived-experience with suicide recovery
3. actively contribute to PLF-PE session discussions
• discuss coping strategies they use
• share their experience with contacting the crisis line
• explain any experience with urge restriction
• identify their warning signs and risk behaviors
• share how they build friendships
• model asking for help
• relate how treatment adherence is helpful
• describe what “hope” means to them
• comment on how PLF has aided them in their recovery
• discuss how they use their safety plan
• share their reasons for living
4. Peer models homework completion
5. Peer facilitates role play exercises by going first
6. Peer facilitates the discussion on firearm safe storage by sharing what steps they have made and why

## 4 Methods

### 4.1 Setting and sample

A clinician, a PRL from WAT22, and four PLF-PE Veteran group members who were WAT22 first responders participated in this community case study. Participants attended 8.8 of 10 sessions of PLF-PE on average and completed post-group qualitative interviews.

### 4.2 Qualitative methods

Qualitative approaches have been successfully applied in previous studies of suicidal individuals’ views and was selected to investigate group participants’ views on acceptability, feasibility, and impact of the PLF-PE intervention. All interviews were conducted via Webex, a HIPAA-compliant video conference platform. Interviewers had bachelor’s and master’s degree in clinical psychology or mental health counseling and received training in qualitative interviewing (e.g., role-playing) and biweekly supervision (e.g., audio recording review and feedback) by a researcher with expertise in qualitative methods and implementation science (SRP). All interviews were conducted using interview guides and were audio-recorded and transcribed verbatim by a team of research assistants, de-identified, and reviewed for accuracy by the research assistant team.

#### 4.2.1 PLF-PE participant interviews

Participant interview guides were developed using a theoretical framework of acceptability and feasibility of healthcare interventions (30) and revised based on feedback from the PLF-PE research team. We focused on PLF-PE acceptability (e.g., experience in a group with a PRL and clinician and satisfaction), feasibility (e.g., challenges with participation in PLF-PE, experience with telehealth-delivered group intervention) impact (e.g., helpfulness of PLF-PE, enduring need for addition support or therapy) and recommendations to improve PLF-PE. Examples of questions from the participant interview guide include:

1. “Please describe your experience in a group with a clinician and PRL. What are some of the pros and cons of having the PRL involved in this group?”
2. “What did you like most/least about PLF-PE? What made it easy/hard to participate in PLF-PE?”
3. “In what ways did PLF help or not help you?”
4. “What impact did the PRL have on lethal means (e.g., access to medications, how you store firearms)?”
5. “What recommendations do you have to improve PLF-PE?”

#### 4.2.2 PLF-PE peer recovery leader and PLF clinician interviews

Clinician and PRL interview guides included similar questions about acceptability and feasibility as the participant interviews and included questions informed by the Consolidated Framework for Implementation Research [CFIR; (31)]. The CFIR is an implementation framework to guide understanding of key determinants that impact the implementation of evidence-based practices. We used CFIR constructs relevant to this early stage of PLF implementation with peer recovery leaders. Examples of questions from the clinician and peer interview guide include:

1. “Please describe your overall experience with PLF-PE. What do you think makes PLF-PE successful or not?”
2. “Please describe your role in PLF-PE. How satisfied were you with how the PLF-PE group went?”
3. “What are some barriers you experienced with implementing the PLF-PE group? What made it easy to implement PLF-PE?”
4. “How well do you think PLF-PE meets the needs of suicidal Veterans?”
5. “What was you experience like working with a clinician/peer who delivered this group intervention?”
6. “What are some of your reservations about being a peer recovery leader in PLF-PE?”
7. “What type of training or additional resources would you recommend to another peer considering the role of recovery leader in PLF-PE?”
8. “What recommendations do you have for clinicians working with peer recovery leaders in PLF-PE?”
9. “What types of training or supports do clinicians and peer recovery leaders working together in PLF-PE?”

### 4.3 Qualitative analysis

Interview data were coded by two independent coders (RA team member and SRP). A summary template and matrix analysis approach, a deductive approach to synthesizing interview data into a template of key themes (32), was used to categorize participant responses along key topics (e.g., acceptability, satisfaction, feasibility, suggestions for improvement, and CFIR domains). First, the interviewers summarized the content of participant interviews discussed along key interview topics. Second, a researcher with expertise in implementation science and qualitative methods (SRP) reviewed each summary with each interviewer. Third, the researcher developed a draft template for documenting content emerging from each interview. Column headings represented key topics with some additional categories (e.g., experience with PLF-PE, experience of PLF with PRL, roles and recommendations for the PRL, firearm storage behavior). The coders met regularly after extracting information from 1-2 summaries to review, resolve conflicts in, discuss, and refine the matrix. We used several strategies to maximize the rigor of our qualitative approach including progressively reducing the data using a series of defined steps (e.g., transcribing, summarizing, charting); using multiple team members at each step in data analysis; conducting frequent debriefing meetings throughout data analysis; and keeping an audit trail (33).

## 5 Results

A total of six qualitative interviews were conducted with four PLF-PE Veteran participants (P), one PLF clinician (C), and one Veteran PRL. The three major themes, experience with PLF-PE, PLF-PE implementation (sub-themes include the importance of the peer recovery leader dyad, impacts and consideration of the PRL role, and barriers to PLF-PE), and changes in firearm storage behavior, are reported below.

### 5.1 Experience with PLF-PE

PLF-PE Participant (P), peer recovery leader (PRL) and clinician (C) interviewees all reported positive experiences with PLF-PE and benefits from group participation including increased coping, improved relationships, and changes in firearm storage. A universal theme mentioned in all interviews was the powerful impact of mutual self-disclosure and the group’s most significant element. One participant reflected:

“I realized I wasn’t alone. There’s no amount of money that you could put on … thinking you’re an individual and no one understands you … and then you actually hear a lot of people are going through the same thing, deal with it the same way, feel the same way … it’s reassuring” (P).

Additionally, all Veterans agreed that the ability to deliver PLF-PE via telehealth facilitated the accessibility of PLF and increased participation among group members. They noted that while an in-person modality may have promoted more intergroup connection, it would have been impossible due to the multiple hours of transportation required for attendance, as group participants lived across the state of Arkansas.

PLF-PE participants and the PRL also indicated that participating in vulnerable discussions with fellow Veterans was the most challenging yet most rewarding aspect of the intervention, with participants expressing how profound it felt to “dig deep” (P) and discuss their shared lived experiences. PLF-PE participants also highlighted the group’s flexibility as a key strength of the intervention.

### 5.2 PLF-PE implementation

#### 5.2.1 Importance of clinician/peer dyad

The clinician, PRL, and PLF-PE participants all highlighted the importance of having both a clinician and lived experience peer guide the intervention. The PRL facilitated trust and promoted self-disclosure, sharing and vulnerability among group members, which enhanced group participation. When describing the PRL, one PLF-PE participant stated he.

“helped us open up and helped bridge the gap. I don’t know anybody else that would’ve been like “Yeah, I want to go first” (P).

Another key benefit of the clinician-lived experience peer recovery leader dyad was to serve as a peer bridger between the clinician and other Veteran PLF-PE group members. Distrust towards the VA and hesitancy to work with non-Veteran providers was identified as a prevalent deterrent from seeking mental health treatment among rural veterans, with one Veteran sharing that it was often challenging to “bridge the gap between the Veteran and the civilian” (P) when working with healthcare professionals. The integration of a PRL bolstered group members’ trust in the clinician. One participant stated that the PRL.

“brings legitimacy to the whole thing … [PRL] being able to say ‘I’ve done this. This helps me, it helped me in the past, I’m excited about doing it again’, [the clinician] could say that … but when [PRL] says it, it just means more because he is a Veteran” (P).

PLF-PE participants described the PRL as a “translator” for the clinician, helping PLF-PE Veterans better understand treatment material and clinical concepts through examples from their own lived experience.

Congruent with PLF-PE participants’ experiences, the clinician valued the PRL initiating self-disclosure of lived experiences, noting this as a particularly potent facilitator to group conversation compared to the clinician utilizing examples - as is done in regular PLF without a PRL. The clinician stated.

“without the peer recovery leader, I might bring in examples from other Veterans who’ve gone through the program to illustrate the point, but actually having it be authentic and real from his experience, I didn’t have to do that and it was more powerful because it came from his words and he could elaborate” (C).

In addition to self-disclosure of personal experiences, the clinician valued the PRL’s ability to express support to fellow group members, enhancing interconnectedness and trust between Veterans. The PRL.

“was able to offer support and validation in a really meaningful way that that I think is also part of the magic juice of this intervention and started Veterans caring about one another … [PRL] would just say ‘oh my god, that just sucks’ or ‘wow, I can’t believe that, how hard that must have been’. Those kinds of comments really add to the cohesion … [PRL] was able to carry some of that validation and support in a way that I think [participants] heard in a very meaningful way and is different than how I would have run it by myself” (C).

#### 5.2.2 Impacts and considerations of the peer recovery leader role

In addition to facilitating group cohesion, a key aspect of the PRL role was supporting outreach efforts and retaining participation in the group. As an established member of WAT-22, the PRL actively increasing awareness of PLF-PE among the organization’s members and facilitated referral of potential participants.

The PRL emphasized the importance of being comfortable with being vulnerable with fellow Veterans. He underscored being able and willing to “lay it all out and open” (PRL) during group sessions, noting that Veterans respond to sincerity with respect. Trust between group members and the PRL was felt to be essential for group participation and also served to increase trust in the clinician by association.

Clinician feedback explored recommendations for clinicians who may work with a Veteran PRL in future iterations of PLF-PE including the requirement that Veteran PRLs attend a prior PLF-PE cycle as a participant. This provides exposure to the course material and allow the clinician to assess the peers’ clinical capacities such as “ability to disclose” (C) and to “interact with other peers” (C) while assuring that they are “further along” (C) in their own recovery.

#### 5.2.3 Barriers of PLF-PE

Qualitative interviews highlighted some challenges to implementing PLF-PE. Barriers identified by the clinician, PRL, and PLF-PE participants related to technology (i.e., video conferencing platforms, audio difficulties, or lack of stable and reliable internet access). Interviewees also reported that the participation of some PLE-PE participants without video often led to less robust participation with possible hinderance on group cohesion. Concern that participation without video could potentially impact engagement and decrease the clinician’s ability to visually assess participant safety was discussed.

### 5.3 Changes in firearm storage behaviors

All PLF-PE participants were firearm owners with multiple guns in their home. The PRL played an important role in firearm safety discussions by describing his decision to store his firearms more securely. Thus, disclosure enhanced the group’s reception of the firearm content. Reflecting on this, the clinician explained.

“his ability to talk about his own firearm suicide attempts, his recovery paths, it really does pave the way for others to feel comfortable talking” (C).

This comfort would be less easily achieved in a traditional PLF group being solely led by a non-firearm owning civilian clinician. The PRL also played a role in educating the research team and clinician on firearm terminology, use, and storage practices employed by Arkansas Veterans. The peer advised on how to enhance firearm cultural competency including how to broach conversations with participants in ways that would be well received.

The PRL and 75% of the Veterans reported changing access to their firearms as a result of participating in the group. One participant “removed all the weapons [from the home] and put them at [their] son’s house” (P). Another reported moving their guns from unlocked drawers in their home to a lock box inside their locked car trunk to increase the distance between themselves and their firearm. One participant noted that concern expressed by fellow group members was a particularly salient motivator of change, stating:

“I did take the clip out of my firearm because it seemed like the other members were a little concerned and didn’t think that a gun lock was enough, and maybe it wasn’t” (P).

## 6 Discussion

This case study described the successful piloting of a Veteran “peer enhancement” to a virtual suicide safety planning group intervention targeting rural Veterans with elevated suicide risk. Understanding ways to promote engagement and enrich telehealth-delivered suicide prevention services is critical for rural populations where access to care is more limited. Moreover, developing effective strategies to discuss and foster safer storage of firearms, particularly at times of heightened suicide risk is especially relevant in Veteran communities where firearm ownership rates are elevated, and less effective storage habits may be prevalent. Our novel, manualized 10-session safety planning and firearm safety group approach augmented by involvement of a Veteran PRL led to detailed conversations of how to limit suicide risk, heighted self-disclosure about suicide symptoms, improved connection, enhanced help-seeking behavior, and safer firearm storage. We are unaware of any other published peer-based intervention to date that includes both a clinician and a lived experience peer coordinating in a group setting.

### 6.1 Lessons learned

1. Virtual group psychotherapy with at-risk suicidal Veterans is feasible, safe, and can yield favorable results. However, some strategies can further augment outcomes.
2. The impact of a safety planning group psychotherapy delivered virtually is significantly enhanced with a lived experience peer (titled “Peer Recovery Leader”) who can facilitate engagement, disclosure, and promote group connection.
3. A local Veteran PRL serves as an important bridge between the clinician and other group members fostering trust, translating clinical material, and promoting access to regional resources.
4. The Veteran PRL is highly influential in advocating for more secure firearm storage practices and enhanced use of the suicide safety plan as a trusted communicator (29) of the value of these practices.

### 6.2 Practical suggestions

1. The PRL receives training in the Project Life Force method, suicide prevention basics and collaborates with the clinician to outline role expectations in advance.
2. The clinician manages any clinical risk and all assessments of suicide symptoms. Clinician and PRL roles must be clearly articulated.
3. Careful consideration of the PRL’s comfort and reactions is necessary, which can be managed with supervision after sessions.
4. The PRL needs to be secure with disclosure of their personal suicide and firearm histories, and honest with the clinician about any reactions with sharing this material.

### 6.3 Future applications

This community case study underscores the importance of leveraging peer support in the service of Veteran suicide prevention. While multiple avenues of peer support exist (17), our novel approach of combining a clinician and lived experience peer together in a group setting is another promising strategy to explore and further develop. This approach is particularly germane with lethal means safety and firearm storage discussions, as government distrust coupled with political arguments regarding second amendment rights complicate VA clinician’s efforts to engage in these discussions. Lived experience PRLs with firearm suicide attempt histories may be particularly effective in facilitating these discussions and next step recommendations are to leverage these capabilities in innovative ways.

## 7 Methodological limitations

Our community case study reported on a telehealth group delivered in rural Arkansas in collaboration with a single community partnership, WAT22. This study only reported qualitative data on six participants, which limits generalizability to other VA-Community partner groups. However, it still provides practical suggestions for groups considering partnered efforts. While other rural areas will have different community partners, this could impact recruitment efforts for PLF-PE groups.

## Data Availability

The raw data supporting the conclusions of this article will be made available by the authors, without undue reservation.
